# Viable gut bacterial metrics associated with intestinal eubiosis and dysbiosis

**DOI:** 10.1080/29933935.2026.2646054

**Published:** 2026-03-21

**Authors:** Tomoaki Naito, Hiromi Setoyama, Noriko Kato-Nagaoka, Tatsuichiro Shima, Tadashi Sato, Kaoru Moriyama-Ohara, Sohei Arase, Takashi Kurakawa, Misato Horino-Higuchi, Saya Tajima, Tomomi Suzuki-Ohnari, Yoshimi Aiyama-Suzuki, Taeko Hara, Eiichiro Naito, Nami Hayashi, Kozo Tsuruta, Tetsuji Hori, Akito Kataoka-Kato, Ryoko Fukuda, Keiichi Mitsuyama, Satoshi Matsumoto, Hirokazu Tsuji

**Affiliations:** a Yakult Central Institute, Tokyo, Japan; b Benesse Institute for Research on KAIGO (Continuing Care), Benesse Style Care Co., Ltd., Tokyo, Japan; c Division of Gastroenterology, Department of Medicine, Kurume University School of Medicine, Fukuoka, Japan; d Department of Gastroenterology, Inflammatory Bowel Disease Center, St. Mary's Hospital, Fukuoka, Japan

**Keywords:** Viable gut microbiota, bacterial viability, calprotectin, *Bifidobacteriaceae*, *Lachnospiraceae*

## Abstract

Gut microbial turnover in humans remains poorly understood. We evaluated gut bacterial viability by quantifying viable cells and relating them to indicators of gut environmental status. Fecal microbial loads (microbial cells per gram feces) were quantified by flow cytometry using the DNA-intercalating dye SytoBC, and viable bacteria were identified by fluorescence in situ hybridization with the 16S rRNA-targeted Eub338 probe. Bacteria retaining sufficient rRNA were defined as viable, and the living bacterial rate was calculated as the proportion of Eub338-positive cells among SytoBC-positive cells. These metrics were applied to starvation‑cultured gut bacterial strains and fecal samples from patients with ulcerative colitis (UC), older adults, and healthy individuals. Starvation culture showed time‑dependent decreases in living bacterial rate and viable cell counts, consistent with colony-forming unit measurements. Patients with active UC exhibited reduced fecal microbial loads and viable microbial loads. Living bacterial rates negatively correlated with fecal calprotectin and Mayo score. In older adults, reduced living bacterial rates and viable *Bifidobacteriaceae* were associated with higher fecal calprotectin. In healthy individuals, lower living bacterial rates and viable *Lachnospiraceae* were associated with harder stools and reduced defecation frequency. Quantitative profiling of viable fecal bacteria may aid in characterizing intestinal homeostasis and dysbiosis‑associated conditions.

## Introduction

The terms eubiosis and dysbiosis are generally used to describe the state of the gut microbiota. Eubiosis refers to a healthy, balanced microbiota, whereas dysbiosis indicates an abnormal, disturbed microbial state.^1-3^ The gut microbiota plays a pivotal role in human health, and shifts from eubiosis to dysbiosis have been linked not only to digestive disorders but also to extra-intestinal diseases (including encephalitis, multiple sclerosis, Parkinson's disease, and hepatitis).^4-8^ Several indicators are commonly used to determine the state of eubiosis or dysbiosis, including microbial diversity and compositional alterations in the intestinal microbiota, pathogenic microorganism detection, and variations in metabolites, such as short-chain fatty acids (SCFAs), secondary bile acids, trimethylamines, tryptophan metabolites, and polyphenols.^1-3^ Recently, fecal microbial loads (microbial cells per gram of feces) have gained attention as an important indicators of the intestinal environment, as it varies across enterotypes and is reduced in conditions such as Crohn's disease.^9^^,^^10^ Furthermore, multiomics approaches, including metagenomic and metatranscriptomic analyses using next-generation sequencing have advanced our current understanding of dysbiosis at the genomic level.^11^^,^^12^ Despite these advances, the boundary between eubiosis and dysbiosis, as well as the underlying mechanism driving dysbiosis, remains incompletely understood, highlighting the need for new perspectives.

Host cells undergoing necrosis or programmed necrosis release damage-associated molecular patterns (DAMPs), such as high mobility group box1, S100 proteins, including calprotectin, ATP, nucleic acid, and mitochondrial DNA that interact with host pattern recognition receptors (PRRs), thereby activating the innate immune system and amplifying inflammatory responses.^13^ In bacteria, cell death occurs through various mechanisms, such as bacteriolysis or bacterial fragmentation, leading to the release of components from dead bacteria, typically microbe-associated molecular patterns (MAMPs) and pathogen-associated molecular patterns (PAMPs).^14^^,^^15^ These microbial components also activate various innate immune cascades via PRRs.^16-18^ In the gastrointestinal tract, a continuous cycle of gut microbial turnover is maintained by a complex interplay of internal factors (e.g. microbial interactions, host digestive enzymatic factors, and antimicrobial peptides) and external (e.g. diet and medication) factors.^19-25^ Destructive death of intestinal bacteria and the consequent release of substantial quantities of MAMPs and PAMPs can adversely impact host health.^26-28^ For example, administration of ampicillin to mice for 2 weeks reduced microbial community diversity and increased gut permeability, accompanied by increased serum levels of D-lactose and lipopolysaccharide (LPS).^26^ SARS-CoV-2 infection induces gut microbiome dysbiosis in both mice and humans.^27^ Notably, patients receiving antibiotic treatment during hospitalization show severe microbiome injury, characterized by loss of diversity and depletion of anaerobic taxa.^27^ Moreover, ATP released by intestinal bacteria limits the production of protective IgA against enteropathogens, whereas ATP depletion enhances high-affinity IgA responses.^28^ Collectively, these findings suggest that increased bacterial death and the resulting accumulation of MAMPs and PAMPs can have substantially compromise host health. Components released from dead bacteria—such as ATP, nucleic acids, and intracellular proteins—may act similarly to host-derived DAMPs, further exacerbating inflammatory responses. However, the dynamics of intestinal bacterial survival and death during dysbiosis and eubiosis remain to be elucidated.

Monitoring live microorganisms has traditionally relied on culture methods, reverse transcription-quantitative polymerase chain reaction (RT‒qPCR), and fluorescence in situ hybridization (FISH).^29-31^RT‒qPCR and FISH target specific bacterial rRNA.^29-31^ Notably, FISH-based bacterial counts correlate well with viable counts determined by colony-forming units (CFU) measurements and RT‒qPCR, particularly in studies of intestinal bifidobacteria.^32^ RT‒qPCR offers high sensitivity for detecting specific bacterial taxa, whereas FISH provides a comprehensive overview of bacterial communities. However, RT‒qPCR requires numerous primers to capture the full diversity of intestinal bacteria, and FISH is challenging to quantify. In addition, culture-based methods are limited by medium selection and are unsuitable for rapid and accurate assessment of the bacterial viability. To overcome these limitations, we revisited a strategy that integrates the FISH with flow cytometer (CytFISH method), a platform widely used for detecting particles such as eukaryotic cells and microorganisms. Using the panmicrobial Eub338 probe targeting 16S rRNA, we quantified the fecal living bacterial rate (percentage of viable cells in fecal microbial populations) and viable microbial loads (viable cells per gram).^31^^,^^33^

In this study, we applied these quantitative metrics to evaluate viable gut bacterial profiles in patients with ulcerative colitis (UC), as well as in nonclinical groups, including older individuals and healthy adults. We further examined the potential associations between variations in viable bacterial profiles and physiological indicators, including inflammatory markers and bowel habit-related measures.

## Results

### Quantification of fecal Eub338⁺ bacterial rate and microbial loads using the CytFISH method

**Figure 1.**
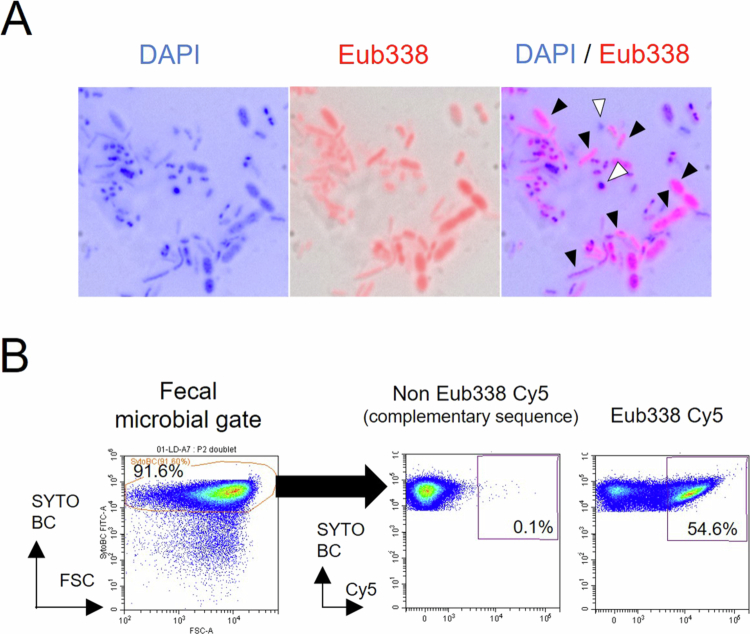
Assessment of living bacterial rate by FISH‑based microscopy and flow cytometry. (A) FISH microscopy images of human fecal samples stained with a Cy5-labeled universal bacterial probe (Eub338; red). Bacterial nucleoids were stained with 4ʹ,6-diamidino-2-phenylindole (DAPI) (blue), and images were merged with bright-field views. Black arrowheads indicate the Eub338^+^ and DAPI^+^ double-positive bacteria, whereas whited arrowheads indicate Eub338-negative Eub338^−^ DAPI^+^ bacteria. (B) Representative flow cytometric gating strategy for quantifying Eub338^+^ SYTOBC^+^ microbial fractions in human fecal samples. The middle panel shows the control stained with a Cy5-labeled non-Eub338 probe.

We first performed microscopic observations using the DNA intercalator 4ʹ,6-diamidino-2-phenylindole (DAPI), a nonspecific DNA intercalating dye, and the panbacterial FISH probe Eub338, designed using 16S rRNA. In healthy fecal samples from healthy adults, abundant Eub338⁺DAPI⁺ bacteria were observed, whereas DAPI-only bacteria constituted a relatively small portion (Figure 1A). To further quantify viable bacterial populations, we employed the CytFISH approach, which integrates FISH with flow cytometry (FCM). In this method, total bacterial populations were identified using SytoBC, a membrane‑permeable DNA‑binding dye that stains both live and dead bacteria and is widely used for enumerating microbial cells by FCM.^34^ Using the 16S rRNA‑targeted Eub338 probe, we measured the proportion of Eub338‑positive bacteria within SytoBC‑positive fecal bacterial populations (Figure 1B). Because the Eub338 probe binds to 16S rRNA, a reduction in its fluorescence intensity was interpreted as degradation of 16S rRNA, indicating that the bacterium is close to death despite retaining its cellular structure and DNA. Bacteria exhibiting fluorescence intensity greater than that of the complementary control probe—reflecting the presence of residual rRNA—were regarded as “viable bacteria.” The proportion of these bacteria within the SytoBC‑positive population was defined as the “living bacterial rate” (Figure 1B). These observations show that CytFISH can visualize differences in bacterial rRNA content and reliably quantify viable bacterial fractions.

### Eub338⁺ bacterial fractions decline under conditions that reduce bacterial viability

**Figure 2.**
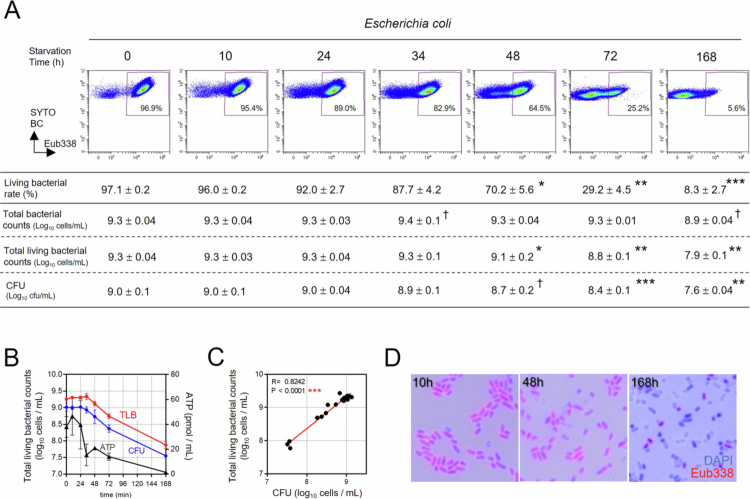
Relationship between Eub338-positive (Eub338^+^) bacteria and colony-forming units (CFU) in an *in vitro* starvation culture model. (A) Representative flow cytometric data showing temporal changes in the percentage of Eub338^+^ cells (living bacterial rate), total bacterial counts, total living bacterial counts, and CFU in *Escherichia coli* cultured under starvation conditions. Values were calculated from triplicate measurements at each time point and are presented as the mean ± SD. Statistical analysis was performed using Dunnett's multiple comparison test comparing 0 h with each subsequent time point. ^†^*p* < 0.1, *p < 0.05, **p < 0.01, and ***p < 0.001. (B) Temporal changes in total living bacterial counts (TLB), CFU, and adenosin triphosphate (ATP) levels in *E. coli* culture supernatant. (C) Spearman's correlation analysis between total living bacterial counts and CFU in *E. coli* culture (***p < 0.001). (D) FISH images of *E. coli*cultured for 10, 48, and 168 h under starvation conditions, stained with a Cy5-labeled Eub338 probe (red). Bacterial nucleoids were stained with 4ʹ,6-diamidino-2-phenylindole (DAPI) (blue).

Subsequently, to verify whether the living bacterial rate calculated by CytFISH accurately reflects bacterial viability, we assessed CFU counts together with the total living bacterial counts calculated by multiplying total bacterial counts by living bacterial rates using intestinal bacterial strains and a starvation culture system. Previous studies have shown that FISH-based bacterial counts closely match CFU measurements, supporting the validity of our approach.^32^ To determine how changes in the proportions of viable and dead bacteria within the same sample are reflected in CytFISH‑based measurements, we examined the relationship between CFU counts and CytFISH-derived numerical profiles under a starvation culture model. Starvation led to a clear time-dependent reduction in the living bacterial rates of *Escherichia coli* (Figure 2A). Although total *E. coli* counts (bacterial cells per mL) showed little change (from 9.3 to 8.9 log_10_ cells/mL), living *E. coli* counts (viable cells per mL) markedly declined (from 9.3 to 7.9 log_10_ cells/mL), consistent with the reduction in CFU (from 9.0 to 7.6 log_10_ cells/mL) (Figure 2A). This transition was accompanied by adenosine triphosphate (ATP) release into the culture supernatant and a decrease in Eub338^+^ bacterial counts (Figure 2B–D). Five representative gut bacterial strains (*Phocaeicola vulgatus*, *Bacteroides uniformis*, *Dorea formicigenerans*, *Clostridium leptum*, and *Bifidobacterium longum*) were also subjected to nutrient deprivation. As observed in *E. coli*, starvation cultures of all five strains demonstrated a decrease in living bacterial rates, minimal or no change in bacterial counts (bacterial cells per mL), and close agreement between living bacterial counts (viable cells per mL) and CFU values (Supplemental Figure 1A–C, Supplemental Table 1). Furthermore, fecal samples collected after antibiotic intake showed marked reductions in both the microscopic appearance and proportion of Eub338⁺ bacteria, suggesting that the living bacterial rate is sensitive to antibiotic‑induced loss of viability (Supplemental Figure 2). Collectively, these findings indicate that total bacterial counts comprise a mixture of viable and dead bacterial cells, whereas living bacterial rates and living bacterial counts reflect viable bacteria capable of growth *in vitro*. The reduction in Eub338⁺ bacteria after antibiotic exposure further suggests that these CytFISH-derived metrics reliably capture the viable bacterial fraction in the intestinal environment.

### Fecal quantitative profiles are reduced in active UC

**Figure 3.**
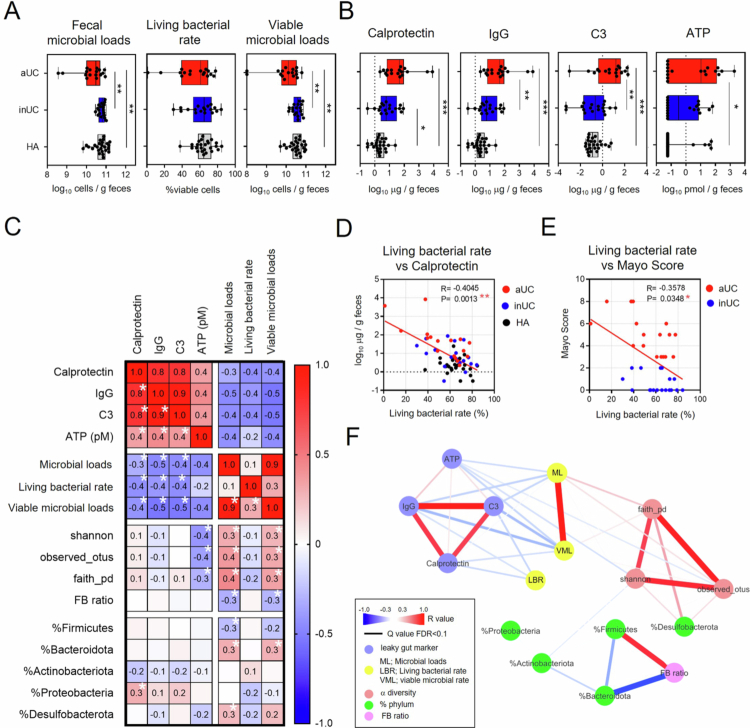
Relationship between dysbiosis-related parameters and fecal microbial loads and viability in patients with ulcerative colitis (UC). (A) Quantification of fecal microbial loads, living bacterial rates, and viable microbial loads in patients with active UC (aUC), inactive UC (inUC), and healthy adults (HA). Statistical analysis was performed using the Kruskal‒Wallis test with Dunn's multiple comparison post-test (*p < 0.05, **p < 0.01, and ***p < 0.001). (B) Quantification of intestinal permeability indicators, i.e. calprotectin, IgG, complement 3 (C3), and adenosin triphosphate (ATP), in fecal supernatants from patients with aUC, inUC, and HA. Statistical analysis was performed using the Kruskal–Wallis test followed by Dunn's multiple comparison post-test (*p < 0.05, **p < 0.01, and ***p < 0.001). (C) Spearman's correlation analysis of intestinal permeability-related indicators, fecal microbial loads, living bacterial rate, viable microbial loads, α-diversity, Firmicutes/Bacteroidota (FB) ratio, and relative abundances of fecal bacterial phyla, in samples from patients with aUC, inUC, and HA with Benjamini–Hochberg false discovery rate correction (*p < 0.05). (D) Spearman's correlation analysis of living bacterial rate and fecal calprotectin levels in patients with aUC, inUC, and HA. (E) Spearman's correlation analysis of living bacterial rate and Mayo scores in patients with aUC and inUC. (F) Spearman's correlation network analysis based on the variables shown in panel C.

CytFISH analysis was first applied to fecal samples to quantify the proportion of Eub338⁺ bacteria. In healthy adults, the proportion of Eub338⁺ bacteria was relatively stable, with a median of 68.1%, a mean of 67.4%, and a standard deviation of 8.7% (Figure 3A). We then examined the quantitative profiles of living intestinal bacteria in fecal samples from participants with UC, which showed typical features of dysbiosis. Patients with active UC (aUC) exhibited lower fecal microbial loads and viable microbial loads than those with inactive UC (inUC) or healthy adults (HA) (Figure 3A). Fecal supernatants from patients with aUC revealed elevated levels of calprotectin, IgG, complement 3 (C3), and ATP compared with those from healthy individuals (Figure 3B). In fecal supernatants from patients with inUC, calprotectin concentrations were higher than in healthy individuals (Figure 3B). These markers showed strong negative correlations with fecal microbial loads and viable microbial loads (Figure 3C). Additionally, living bacterial rates were negatively associated with these markers as well as with Mayo scores (Figure 3C-E). Conversely, no significant differences were observed between the groups in Shannon or Faith phylogenetic diversity indices, and only the observed features in aUC tended to be lower than those in inUC (Supplemental Figure 3A). Furthermore, no correlation was observed between α-diversity and other dysbiosis markers, except ATP (Figure 3C, F). Analysis of taxonomic abundance revealed a lower relative abundance of Bacteroidota and a higher Firmicutes/Bacteroidota ratio (FB ratio) in aUC than in inUC (Supplemental Figure 3B–C). However, these indices were not correlated with dysbiosis markers such as calprotectin (Figure 3C, F). Network analysis of these relationships demonstrated that calprotectin, IgG, and C3 in fecal supernatants from patients with UC were more strongly associated with fecal microbial loads, viable microbial loads, and living bacterial rates than with α-diversity or bacterial taxon abundance (Figure 3F). Consequently, these findings suggest that the quantitative profile of viable gut bacteria in fecal samples may serve as an indicator of UC disease severity.

### Reduced bifidobacterial viability is associated with impaired intestinal barrier integrity in older individuals

**Figure 4.**
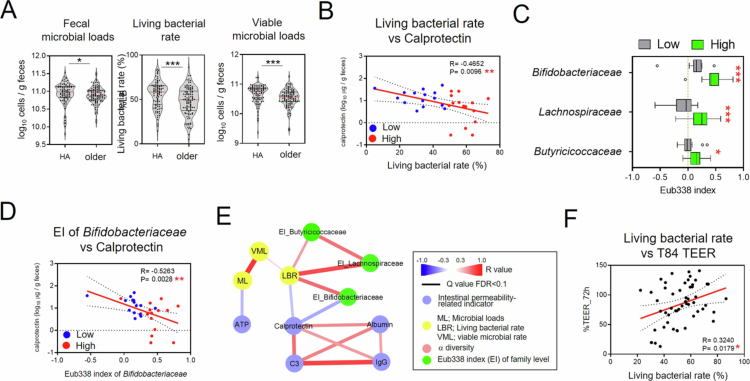
Associations between dysbiosis-related parameters and living bacterial rates in the older population. (A) Quantification of fecal microbial loads, living bacterial rate, and viable microbial loads in healthy adults (HA) and older participants. Statistical analysis was performed using the Mann–Whitney *U* test (*p < 0.05 and ***p < 0.001). (B) Spearman's correlation analysis between living bacterial rate and the fecal calprotectin concentrations. Blue dots indicate participants with a low living bacterial rate (below the median value, <49.57%), whereas the red dots indicate those with a high living bacterial rate (≥49.57%). (C) Eub338 index (EI) analysis of fecal bacteria at the family level in older participants with high versus low living bacterial rates. Statistical analysis was performed using the Mann–Whitney *U* test with Benjamini–Hochberg false discovery rate correction (*p < 0.05 and ***p < 0.001). (D) Spearman's correlation analysis between the EI of *Bifidobacteriaceae* and the fecal calprotectin concentration. (E) Correlation network showing Spearman's correlation analysis of fecal microbial loads, living bacterial rates, viable microbial loads, Shannon diversity, intestinal dysbiosis-related indicators, and the EI of *Bifidobacteriaceae*, *Lachnospiraceae*, and *Butyricicoccaceae* with Benjamini‒Hochberg false discovery rate correction (*p < 0.05). (F) Spearman's correlation analysis between the living bacterial rate in fecal samples from older participants and transmembrane epithelial electrical resistance (TEER), measured in the T84 epithelial monolayers stimulated with fecal supernatant from older participants.

Next, we investigated the relationship between the gut environment and viable bacteria in the older individuals, a well-studied case of dysbiosis.^35-37^ The geriatric population generally comprises individuals in good health as well as those experiencing age-related health problems. Therefore, we hypothesized that the gut microbiota in older individuals fluctuates between eubiosis and dysbiosis. Consistent with this hypothesis, our analysis showed that older individuals exhibited lower fecal microbial loads, living bacterial rates, and viable microbial loads than healthy adults (Figure 4A). Notably, the living bacterial rate was negatively correlated with fecal calprotectin levels (Figure 4B, Supplemental Figure 4), suggesting that the reduced living bacterial rates in older individuals were associated with dysbiosis. These observations prompted us to examine the characteristics of living fecal bacteria in older individuals with low and high living bacterial rates. We therefore divided the older participants into two groups based on the median living bacterial rates (49.6%) and compared the Eub338 index of fecal bacterial families between the groups with high- and low-living-bacterial-rate groups. We found that the older individuals with low living bacterial rates showed lower Eub338 indices of *Lachnospiraceae*, *Butyricicoccaceae*, and *Bifidobacteriaceae* than those with high living bacterial rates (Figure 4C). Subsequently, we evaluated the relationship between intestinal permeability markers, which indicate changes in epithelial barrier integrity and paracellular permeability, and viable bacteria in older individuals. A negative correlation was observed between calprotectin concentration in fecal supernatants and the Eub338 index of *Bifidobacteriaceae* (Figure 4D, E). These findings suggest that fecal supernatants from older individuals with low living bacterial rate may contain factors that impair intestinal barrier integrity. Furthermore, we assessed epithelial permeability using T84 cells, a human colonic epithelial cell line widely used as an *in vitro* model of intestinal barrier function.^38^ Consistent with this interpretation, transepithelial electrical resistance (TEER) in T84 cells was positively correlated with the living bacterial rate (Figure 4F). Taken together, the viability of intestinal bacteria, particularly *Bifidobacteriaceae*, may be associated with the status of the intestinal mucosal barrier in older individuals. This relationship may reflect a characteristic feature of dysbiosis in aging populations, although its biological significance remains to be clarified.

### Living bacterial rate and viable Lachnospiraceae are associated with abdominal and defecation symptoms in healthy adults with mild constipation

**Figure 5.**
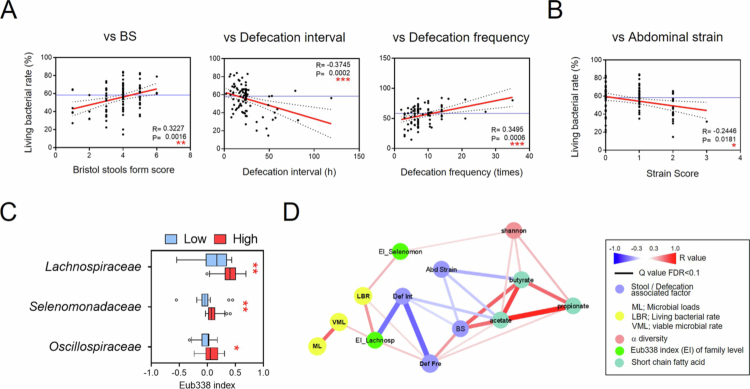
Associations among defecation patterns, abdominal symptoms, and living bacterial rate in healthy adults with mild constipation (HA). (A) Spearman's correlation analysis of living bacterial rates and defecation-related symptoms, i.e. Bristol stool form score (BS), defecation interval, and defecation frequency in HA. (B) Spearman's correlation analysis between living bacterial rates and abdominal strains in HA. (C) Eub338 index (EI) analysis of intestinal bacteria at the family level in participants with high- (≥59.81%) versus low-living-bacterial rates (<59.81%). Statistical analysis was performed using the Mann–Whitney *U* test with Benjamini‒Hochberg false discovery rate correction (*p < 0.05 and **p < 0.01). (D) Correlation network based on Spearman's correlation analysis of fecal microbial loads, living bacterial rates, viable microbial loads, Shannon diversity, defecation and abdominal symptoms, fecal short-chain fatty acids (SCFA), and EIs of *Lachnospiraceae* and *Selenomonadaceae* with Benjamini‒Hochberg false discovery rate correction (*p < 0.05).

Subsequently, we investigated the importance of monitoring live bacteria during eubiosis in healthy adults. The living bacterial rate was positively correlated with the Bristol stool form score and defecation frequency (Figure 5A), whereas it was negatively correlated with the defecation interval and abdominal distension strain during defecation (Figure 5A, B). These findings suggest that healthy individuals with low living bacterial rates are more likely to experience constipation-related symptoms, including harder stools, reduced defecation frequency, prolonged defecation intervals, and abdominal strain. To identify bacterial traits associated with these symptoms, we stratified the healthy adults cohort according to the median living bacterial rate (59.8%). We then compared the Eub338 index of fecal bacterial families between the high- and low-living-bacterial-rate groups. Individuals in the low living bacterial rate group exhibited lower Eub338 indices for *Lachnospiraceae*, *Selenomonadaceae*, and *Oscillospiraceae* than those in the high-living bacterial rate group (Figure 5C). Notably, the Eub338 index of *Lachnospiraceae* was positively correlated with defecation frequency and negatively correlated with defecation interval (Figure 5D, Supplemental Figure 5). In contrast, SCFAs, such as acetic and butyric acid, were negatively correlated with defecation intervals but not with the Eub338 index (Figure 5D, Supplemental Figure 5). Taken together, these observations indicate that reduced viability of *Lachnospiraceae* in healthy adults with mild constipation is associated with less favorable bowel habits. Although this pattern suggests a link between *Lachnospiraceae* viability and defecation-related symptoms, its physiological relevance remains to be clarified.

## Discussion

We found that fluctuations in living fecal bacteria intrinsically and pivotally reflect host health status. These fluctuations were captured by quantitative profiles, including fecal microbial loads, viable microbial loads, and living bacterial rates. Although these parameters are interrelated, their subtle differences underscore their distinct and unique contributions. Fecal microbial load has been used as an indicator of fecal microbiome characteristics.^39-42^ Vadeputte *et al*. demonstrated that quantitative microbial profiling is indispensable for interpreting microbial features that cannot be inferred from relative abundance alone.^9^ In this study, we performed starvation culture experiments to investigate the relationship between total bacterial counts and bacterial viability. In this context, microbial load represents the total number of intact bacterial particles, including live, dead, and inactive bacteria, whereas living bacterial rates or viable microbial loads reflect the fraction of bacteria capable of growth. Under starvation conditions, total bacterial counts decreased only slightly over time, whereas total living bacterial counts, living bacterial rates, and CFU declined substantially.

Because reductions in microbial loads likely reflect the breakdown of dead bacteria, we hypothesized that starvation cultures would reveal both the loss of viable bacteria and the breakdown of dead and/or inactive bacteria. Supporting this interpretation, extracellular ATP, which is often used as an indicator of bacterial membrane damage due to leakage of intracellular contents, was detected in the culture supernatant.^43^^,^^44^ These observations suggest that, *in vivo*, a decrease in microbial loads may represent a more advanced dysbiotic state than a decrease in living bacterial rates alone, although further mechanistic studies are required to clarify this relationship. In active UC, both microbial loads and viable microbial loads were markedly reduced. Some specimens exhibited microbial loads as low as one thousandth of the average (Figure 3A). In healthy individuals, microbial loads typically range from 10.9 to 11.0 log_10_ cells per gram of feces (Figure 3A).^9^ In inactive UC, microbial loads were only slightly lower than those in healthy individuals; however, in active UC, microbial loads averaged 10^10.5^ cells, which is approximately half of the healthy level. This pattern may reflect a substantial reduction in bacterial presence in the intestinal environment during active inflammation. Because intestinal barrier function is impaired in IBD and enteric bacterial LPS is detectable in patient plasma,^45^^,^^46^ these findings may be partly attributable to the collapse of dead or damaged intestinal bacteria. Calprotectin is released mainly from necrotic neutrophils during inflammation, and to our knowledge, no studies have demonstrated that calprotectin directly affects bacterial viability or interferes with FISH‑based detection.^47^ Therefore, its influence on our viability assessment was considered minimal. Because bacterial metabolites and structural components are essential for maintaining epithelial integrity,^16^^,^^18^^,^^48^ a substantial loss of bacterial biomass could further compromise mucosal function. Thus, a marked reduction in microbial loads may represent a more severe dysbiotic state.

Numerous studies have extensively investigated gut dysbiosis in the geriatric population, encompassing a wide range of health conditions, such as constipation, diarrhea, infections, malnutrition, osteoporosis, sarcopenia, frailty, and dementia.^35^^,^^36^ In this study, the living bacterial rates and the Eub338 index of *Bifidobacteriaceae* were negatively correlated with fecal calprotectin concentrations, indicating that reduced living bacterial rates in older individuals were associated with diminished *Bifidobacteriaceae* viability and impaired intestinal barrier function (Figure 4B–F). These findings suggest that a decline in *Bifidobacteriaceae* viability may be a characteristic feature of ageing enteroenvironments. Previous studies have reported that decreased abundance of *Bifidobacterium* spp. is a hallmark of dysbiosis in older individuals.^35^^,^^36^ Emerging evidence suggests that increasing intestinal *Bifidobacterium* spp. populations may improve health outcomes. For example, supplementation with the prebiotic 1-kestose increases *B. longum* abundance and ameliorates muscle atrophy in extremely old patients with sarcopenia.^49^ Similarly, administration of *B. longum* to aged or cognitively impaired 5 × FAD-transgenic mice improved cognitive function.^50^ These reports may offer insights that could inform future approaches to addressing age‑related reductions in *Bifidobacterium* viability. Understanding the mechanisms that sustain *Bifidobacterium* viability in the gut of older individuals may help clarify the drivers of dysbiosis and ultimately contribute to strategies that promote healthier aging.

A considerable reduction in living bacterial rates was observed during the initial phase of starvation. This observation led us to speculate that fluctuations in fecal bacterial viability may reflect moderate or subtle alterations in the gut microbiota, including shifts between eubiosis and dysbiosis. To explore this possibility, we examined the relationship between the gut microbiota, fecal and abdominal symptoms, and bacterial viability in healthy individuals. We found significant correlations between living bacterial rates and these parameters (Figure 5A, B, D, Supplemental Figure 5). Previous studies have investigated the association between constipation and intestinal bacteria, particularly its relationship with intestinal metabolites.^51-53^ Secondary bile acids, serotonin (5-hydroxytryptamine), and methane, which are produced or modified by intestinal bacteria, are known to influence intestinal peristalsis. ^52^^,^^54^^,^^55^ Constipation is closely linked to cardiovascular and renal diseases, with increased risk attributed to gut microbiota-derived metabolites such as trimethyl-amine-N-oxide, *p*-cresol sulfate, and indoxyl sulfate.^51^ In our study, comparison of individuals with high and low living bacterial rates revealed that *Lachnospiraceae* viability was associated with abdominal and defecation symptoms, particularly defecation interval and frequency (Figure 5C, D, Supplemental Figure 5). This association raises the possibility that reduced *Lachnospiraceae* viability may influence gut metabolite production and contribute to constipation-related symptoms. The *Lachnospiraceae* family includes butyrate- and acetate-producing genera such as *Blautia*, *Coprococcus*, *Dorea*, *Lachnospira*, and *Roseburia.*^56-58^ Reduced viability of these SCFA-producing bacteria in individuals with mild constipation is consistent with previous reports showing decreased fecal acetic acid levels in constipated individuals.^53^ Previous studies have focused on changes in specific bacteria, such as reductions in beneficial microbes (including *Bifidobacterium* and lactic acid-producing bacteria) or increases in opportunistic bacteria in patients with constipation.^52^ Our findings may help generate hypotheses regarding the mechanisms underlying the decline in *Lachnospiraceae* and its potential relevance to constipation. Further studies are required to clarify these relationships and to explore their implications for future therapeutic strategies.

Several approaches have been developed to assess bacterial viability, including propidium monoazide (PMA)-based methods that discriminate between live and dead cells based on membrane integrity.^59^ In contrast, our FISH-based approach identified cells that retained detectable 16S rRNA, indicating preserved metabolic activity rather than membrane permeability. Previous studies have shown good agreement among FISH counts, qPCR values, and CFU measurements for representative gut bacteria, supporting the validity of rRNA-based viability assessment.^32^ However, because PMA and FISH rely on different biological properties, these methods may not yield identical results; rather, each captures a distinct aspect of microbial viability.

We believe that our focus on fecal microbial loads, viable microbial loads, and living bacterial rates provides a useful framework for characterizing the gut environment and identifying factors that may contribute to dysbiosis. Our findings suggest that reduced microbial loads may be associated with disease severity in active UC (Figure 3A). In older adults, decreases in living bacterial rates and viable *Bifidobacteriaceae* are linked to impaired intestinal barrier function, whereas in healthy individuals (Figure 4B, E), reduced *Lachnospiraceae* viability is associated with bowel habit-related symptoms (Figure 5A, D). These observations suggest that an increase in dead bacteria and their components may be associated with more severe dysbiosis; however, further validation is required. When dead bacteria-derived MAMPs and PAMPs, rather than cell surface structures from living bacterial cells-accumulate at high concentrations or persist for prolonged periods in the gut, a vicious cycle may be initiated in which neutrophils and intestinal epithelial cells respond by releasing DAMPs such as calprotectin. Although, this hypothesis requires further investigation, it may represent a potential mechanism underlying dysbiosis. Monitoring the quantitative profile of viable gut bacteria, together with SCFAs and α-diversity, may serve as a useful tool for understanding eubiosis and dysbiosis. Integrating information on bacterial viability with existing microbiome data could enhance analytical accuracy and deepen our understanding of host‒microbe interactions.

A technical limitation of our approach is that FISH requires fixation of stool samples, which precludes the direct assessment of metabolites produced by living bacteria. Although isolating live bacteria under anaerobic conditions would allow direct evaluation of their metabolic activity, such approaches were beyond the scope of this study. Consequently, additional methodological considerations are warranted. Further, probe affinity can vary among bacterial taxa, potentially influencing the absolute estimates of living bacterial rates; thus, FISH may underestimate bacteria with low metabolic activity or overestimate structurally intact but non‑viable cells. Moreover, our approach cannot distinguish bacteria in a viable-but-non-culturable (VBNC) state. Because FISH defines viability based on rRNA integrity, whereas PMA-based methods rely on membrane permeability, these techniques capture different aspects of bacterial viability, and discrepancies between them may occur. Finally, the Eub338 index is an exploratory, nonstandardized metric derived from FISH-based sorting, and its biological interpretation remains preliminary. Some additional limitations of this study should be acknowledged. Cohort sizes were relatively small, which limits the generalizability of the results. Being an observational study, causal relationships between bacterial viability, dysbiosis, and host symptoms cannot be established. Stool collection conditions, diet, and bowel habits may influence microbial viability, and residual confounding factors cannot be excluded. Future studies incorporating larger cohorts, longitudinal designs, and multiple viability-based methodologies are essential to validate and extend our findings. Nonetheless, our results may encourage further exploration of the significance of live bacteria in the gut microbiota and may help advance research on host health, prebiotics, postbiotics, and disease treatments.

In summary, our study shows that quantitative profiling of viable gut bacteria captures meaningful fluctuations in intestinal homeostasis across health, aging, and disease. These findings highlight bacterial viability as an important dimension of dysbiosis and suggest its potential relevance to host symptoms and mucosal function. Future studies integrating viability-based metrics with broader microbiome analyses will be essential to clarify underlying mechanisms and advance therapeutic strategies.

## Materials and methods

### Human subjects

For ulcerative colitis (UC), the experimental protocols were approved by the Ethics Committee of Kurume University of Medicine, Fukuoka, Japan (approval number: 16241). Experimental protocols involving older participants were approved by the Ethics Committee of the Benesse Institute for Research on KAIGO (approval number: 20190401). This study was registered in the University Hospital Medical Information network Research Center public database (UMIN Study ID: UMIN000036684). For healthy adults, the experimental protocols were approved by the ethical committees of the Chiyoda Para Medical Care Clinic (approved number; YKH19C1), Tokyo, Japan, or the Nihonbashi Cardiology Clinic, Tokyo, Japan (approval number; NJI-019-01-01). All study procedures complied with all relevant ethical regulations, adhered to Declaration of Helsinki, and were in accordance with Japan's privacy laws. All participants provided both oral and written informed consent. A total of 35 patients with ulcerative colitis with UC (17 with active UC and 18 with inactive UC) and 25 healthy adults were recruited at Kurume University Hospital, Fukuoka, Japan (Supplemental Table 2). Detailed patients background are provided in Supplemental Table 3. Fecal samples were obtained from 109 older volunteers residing at the Benesse Style Care Nursing Facility from a previously established cohort (Supplemental Table 4).^60^ The healthy adult cohort comprised 93 volunteers working in an office environment (Supplemental Table 4); three individuals who had taken cephem antibiotics accidentally prior to stool collection were excluded from the data of 93 healthy adults. Their stool samples were analyzed separately: subject X (male, 33 y) provided a sample 4 d after antibiotic intake, subject Y (female, 32 y) after 3 d, and subject Z (male, 39 y) after 2 d. Fecal samples from healthy volunteers were obtained from a previously established cohort.^61^ At the time of stool collection, participants completed a brief questionnaire assessing gastrointestinal symptoms and bowel habits, including abdominal distension strain, defecation frequency, and defecation interval, which were used to characterize symptom profiles associated with each fecal sample. For the evaluation of disease activity, clinical activity in patients with UC was graded using the partial Mayo score (inactive disease was defined as a score <2 with no individual subscore >1 point), and active UC was defined as a partial Mayo score ≥3.^62^ Medication status at the time of stool collection for patients with UC is summarized in Supplementary Tables 2 and 3. Stool samples were collected in a plastic receptacle with lids, placed in labeled, nontransparent freeze bags, frozen below −20 °C, transported to the laboratory, and stored at −80 °C until analysis.

### Starvation culture of bacterial strains

For the preliminary culture preceding the starvation culture, the bacterial strains were cultivated under anaerobic conditions at 37 °C until the late logarithmic phase in mGAM broth or mGAM broth supplemented with 1.0% glucose (Supplemental Table 5). *P. vulgatus* and *B. uniformis* were cultivated in mGAM broth for 16 h, whereas *D. formicigenerans*, *C. leptum*, *B. longum*, and *E. coli* were cultivated in mGAM broth supplemented with 1.0% glucose. These cultured bacteria were used as seeds for starvation cultures. Following preliminary cultivation, 1% (w/v) of bacterial growth was inoculated into 20 mL of fresh bacterial culture broth and then culture under anaerobic conditions for 16–24 h. After cultivation, the bacterial culture was centrifuged at 9000 × *g* for 15 min at 4 °C, the supernatant was removed, and the bacteria were washed with phosphate-buffered saline (PBS). The centrifugation and removal of the supernatant were repeated, and the bacteria were resuspended in 20 mL of anaerobic PBS to initiate the starvation culture at 37 °C under anaerobic conditions. Bacterial culture supernatants were collected at 0, 10, 24, 30, 72, and 168 h to assess CFU, total bacterial counts, living bacterial rate, total living bacterial counts, and ATP levels. For CFU, 50 μL of the collected bacterial culture fluid was plated onto GAM agar plates and incubated under anaerobic conditions at 37 °C for 2–3 d. Subsequently, the colonies were enumerated, and CFU were calculated.

### Measurement of total bacterial counts in feces and cultured bacteria

#### Fecal sample preparation

Defrosted fecal samples were diluted ten-fold with PBS and thoroughly shaken with sterile glass beads. The fecal samples were mixed well with three volumes of 4% (wt/vol) paraformaldehyde (PFA)–PBS solution^63^ and left at 4 °C for 16 h. Then, preparations were stored at –80 °C until use.

#### Cultured bacterial sample preparation

Bacterial culture fluid was mixed with three volumes of 4% (wt/vol) paraformaldehyde–PBS solution and maintained at 4 °C for 16 h. Subsequently, the preparations were stored at –80 °C until use.

#### Measurement of fecal microbial load with flow cytometry (FCM)

The PFA-fixed fecal samples were 80-fold diluted with PBS by adding an equivalent volume of PBS. The samples were filtered through a #200 nylon mesh (ASONE, Osaka, Japan) to remove large particles. Furthermore, the fecal suspensions were diluted 400-, 2000-, and 10,000-fold with PBS. Then, each fecal dilution was individually mixed with 10 μL the sample, 10 μL Precision Count Beads (BioLegend [San Diego, CA, USA], 1.03 × 10^6^ particles/mL) for internal standardization, 30 μL PBS, and 150 μL PBS containing 3000-fold diluted SytoBC (Thermo Fisher Scientific, Eugene, OR, USA) in a 96-well plate. The mixtures were incubated on ice for 15–60 min, applied to the CytoFLEX S flow cytometer (Beckman Coulter, Miami, FL, USA), and the fcs data were obtained. The data were analyzed using CytoExpert v2.4 (Beckman Coulter). Fecal microbial loads (cells per gram feces) were determined by calculation from the ratio of the number of SytoBC-positive bacteria to the internal standard bead counts using the following formula: Microbial loads (cells/g feces) = dilution factor × (SytoBC positive counts)/(Beads counts) × 1.03 × 10^6^ (particles/mL). When all data were captured at 100,000 beads by FCM, data in the range of 200 ≤ beads ≤ 1500 total beads acquired were adopted. If there were multiple data points in the dilution series that were within the aforementioned bead range, the value closest to 850, which is the median of the specified range of bead counts, was adopted for the data on microbial load in the fecal samples. As SytoBC‑positive events are detected based on cell size and nucleic acid staining, the quantified microbial population may include not only bacteria but also archaeal cells and small eukaryotic microorganisms of similar size. Large debris and larger eukaryotic cells were excluded during gating; however, microorganisms within the bacterial size range could not be completely distinguished.

### FISH of feces and cultured bacteria

#### Fecal sample preparation

Defrosted feces were diluted with a ten-fold concentration of PBS solution and then vigorously shaken with sterile glass beads (2 mm in diameter). The resulting filtrate was thoroughly mixed with three volumes of a 4% (wt/vol) PFA–PBS solution.^63^ And then left at 4 °C for 16 h. Subsequently, the preparations were stored at –80 °C until use.

#### Cultured bacterial sample preparation

Bacterial culture fluid was mixed with three volumes of 4% (wt/vol) PFA–PBS solution and maintained at 4 °C for 16 h. Subsequently, the preparations were stored at –80 °C until use.

#### Fluorescence labeling method for microscopy

FISH was performed as previously described.^19^^,^^64^ Fixed samples (10 μL) were diluted 100- to 400-fold dilution with cold PBS solution and evenly spread onto a 1-cm^2^ frame on an MAS-coated glass slide (Matsunami Glass ind., Ltd., Kishiwada, Osaka, Japan); the slide was subsequently dried and dehydrated in 96% [vol/vol] ethanol for 10 min. Subsequently, 100 μL of hybridization solution (750 mM NaCl, 100 mM Tris–HCl, 5 mM EDTA, 0.01% [wt/vol] BSA, 0.2% [wt/vol] poly A, 10% [wt/vol] dextran sulfate) containing 450 ng of Cy5- or Cy3-labeled Eub338 [5ʹ-GCTGCCTCCCGTAGGAGT-3ʹ] or Cy5-labeled non-Eub338 [5ʹ-ACTCCTACGGGAGGCAGC-3ʹ] was dropped on the cell smears. The smears were covered with a coverslip, and the glass slide was incubated overnight at 45 °C in a dark box humidified with SET solution (750 mM NaCl, 100 mM Tris–HCl, 5 mM EDTA). Upon completion of the hybridization reaction, the glass slides were incubated for 20 min in a wash solution (50 mM NaCl, 4 mM Tris–HCl, 0.02 mM EDTA) at 50 °C. The slides were then washed again with distilled water and air-dried, and the bacteria were embedded in VECTASHIELD with DAPI (Vector Laboratories, Newark, CA, USA). The slides were then observed under the Leica Q550FW microscope (Leica, Wetzlar, Germany), and images were captured.^64^

### Living bacterial rate assessed by FISH-based FCM (CytFISH)

To prepare the samples for FISH analysis using FCM, 2.0 to 4.0 × 10^7^ cells of PFA-fixed fecal samples were suspended in 500 μL PBS, and the suspension was filtered using a #200 nylon mesh (ASONE) to remove large particles. Next, 500 μL PBS was added, and the suspension was centrifuged at 20,000 × *g* for 5 min at 4 °C. After centrifugation, the supernatant was removed, and the fecal pellet was dehydrated in 96% [vol/vol] ethanol for 10 min at 20 °C. The mixture was centrifuged again, and the supernatant was removed. The pellet was washed with 500 μL of PBS to obtain the bacterial palette. Next, the pellet was hybridized with 100 μL of hybridization solution (750 mM NaCl, 100 mM Tris–HCl, 5 mM EDTA, 0.01% [wt/vol] BSA, 0.2% [wt/vol] poly A, and 10% [wt/vol] dextran sulfate) containing 450 ng of Cy5-labeled Eub338 or Cy5-labeled non-Eub338. The suspension was incubated overnight at 40 °C. After the hybridization reaction, 500 μL of a prewarmed wash solution (50 mM NaCl, 4 mM Tris–HCl, 0.02 mM EDTA) at 45 °C was added to the suspension. The mixture was then centrifuged at 20,000 × *g* for 5 min at 20 °C. The supernatant was removed, and the pellet was resuspended in 1.0 mL prewarmed wash solution and incubated at 45 °C for 25 min. After incubation, the mixture was centrifuged again, and the supernatant was removed. The pellet was washed with 500 μL of PBS and centrifuged at 20,000 × *g* for 5 min at 4 °C. Finally, the pellet was suspended in 350 μL PBS containing 4000-fold diluted SytoBC (Thermo Fisher Scientific). The suspension was then applied to the CytoFLEX S flow cytometer (Beckman Coulter) to obtain fcs data. The data were analyzed using CytoExpert v2.4 (Beckman Coulter). Based on Non-Eub338-Cy3 staining, the Eub338^+^ gate (i.e. the living bacterial gate) was defined based on non-Eub338-Cy3 staining, as shown in Figure 1A. In this study, cells retaining detectable 16S rRNA (Eub338‑positive; Eub338^+^) were operationally defined as viable bacteria, following established FISH‑based viability assessments in previous reports. Viability can also be assessed using propidium monoazide (PMA)-based methods, which rely on membrane permeability rather than rRNA integrity. As these approaches define viability differently, result obtained PMA may not yield be directly equivalent to those obtained using FISH-based cytFISH.

### ATP measurement of bacterial culture supernatant and fecal supernatant

In brief, 100 μL of collected bacterial culture fluid were centrifuged 20,000 × *g* for 5 min at 4 °C, and the supernatant was collected. The preparations were stored at −80 °C until use. To measure ATP concentration, the Kinshiro ATP Luminescence kit ver.2 (FUJIFILM Wako Pure Chemical, Chuo, Osaka, Japan) was utilized following the manufacturer's protocol. The preparations were diluted ten- to 100-fold with MilliQ water, mixed with a luminescence reagent, and incubated for 8–10 min at 20 °C; the luminous flux was measured using the TG-20/20 luminometer (Promega, Madison, WI, USA).

### Enzyme-linked immunosorbent assay (ELISA) of fecal supernatant

Defrosted fecal samples were diluted ten-fold with PBS and vigorously shaken with sterile glass beads (2 mm in diameter). The resulting fecal suspension was centrifuged at 20,000 × *g* for 3 min at 4 °C, and the supernatant was collected. The supernatants were diluted and used for enzyme-linked immunosorbent assays (ELISA). Commercial ELISA sets for human calprotectin (S100A8/A9), human IgG, and albumin were used according to the manufacturers' protocols. To measure human complement 3 (C3), an anti-human C3 antibody (Abcam [Cambridge, UK], ab117244) was diluted 2000 times and plated onto a 96-well plate as the first capture antibody. Subsequently, diluted samples and serially diluted recombinant human C3 (100 ng/mL) were added to 96-well plates. After incubation, a biotin-conjugated anti-human C3 polyclonal antibody was added to the well as the second detection antibody. Subsequently, HRP-conjugated streptavidin was added to the wells, and colorimetry was performed using o-phenylenediamine. The reaction was stopped with 2.5 N sulfate, and the plate was assayed at 492 nm using a iMark microplate reader (Bio-Rad, Hercules, CA, USA).

### Measurement of fecal organic acids

Defrosted fecal samples were diluted ten-fold with PBS and vigorously shaken with sterile glass beads (2 mm in diameter). For protein removal, 450 μL of the resultant fecal suspension was mixed with 50 μL of 10% (v/v) perchloric acid, the mixture was centrifuged at 20,000 × *g* for 5 min at 4 °C, and the supernatant was filtered with a 0.45-μm membrane filter (Millipore, Burlington, MA, USA). The concentrations of organic acids were measured using high-performance liquid chromatography (HPLC) according to a previously reported method.^39^ Fecal organic acids were measured using a standard curve generated with two points (0.01 and 0.1 μmoL/10 μL) of internal standards for each of the following organic acids: sodium salts of acetic acid, butyric acid, isobutyric acid, formic acid, propionic acid, succinic acid, valeric acid, isovaleric acid, and lithium lactate (Kanto Chemical, Chuo, Tokyo, Japan). HPLC was performed using 15 mM perchloric acid and 7% acetonitrile as eluates, 15 mM perchloric acid, 60 mM Tris hydroxymethyl aminomethane, and 7% acetonitrile as pH-adjusting agents, and an Rspak KC-811 (RESONAC [f.k.a. Showa Denko K.K], Minato, Tokyo, Japan) at 42 °C as the elution column. The detector used was a 432 electroconductivity detector (Waters, Milford, MA, USA) with a cell temperature was 45 °C and a flow rate was 1.0 mL/min.

### 16S rRNA gene sequencing and microbial community analysis

Defrosted fecal samples were diluted a ten-fold concentration with PBS and vigorously shaken with sterile glass beads (2 mm in diameter). In brief, 200 μL of the fecal suspension was used for DNA extraction. For extraction, fecal suspension was mixed with 500 μL of extraction buffer [100 mM Tris–HCl (pH 9.0), 40 mM EDTA, 1% SDS (v/v)], 300 mg of sterile glass beads (0.1 mm in diameter, TOMY [Nerima, Tokyo, Japan]), and 500 μL of buffer-saturated phenol (NIPPON GENE, Chiyoda, Tokyo, Japan). The suspension was mechanically disrupted using a FastPrep MP (MP, Chuo, Tokyo, Japan) at a power level of 5.0 for 30 s. Next, the mixtures were centrifuged at 20,000 × *g* at 20 °C for 5 min, and the upper water-soluble fluid was collected in a new tube and mixed with phenol/chloroform/isoamyl alcohol (25:24:1) (NIPPON GENE). The mixture was vigorously shaken with a FastPrep MP at a power level of 4.0 for 45 s and then centrifuged at 20,000 × *g* for 5 min at 20 °C. The upper water-soluble layers were collected in a new tube, and isopropanol was added for DNA precipitation. Finally, the DNA precipitate was suspended in 1 mL of TE buffer. The V1–V2 region of the 16S rRNA gene was amplified from the extracted DNA using the primers 27F mod2-MiSeq and 338R-MiSeq. The PCR cycle was as follows: 50 °C for 2 min; 95 °C for 10 min; and a repeated cycle of 95 °C for 30 s, 55 °C for 30 s, and 72 °C for 90 s. PCR was stopped before signal saturation. The PCR products were purified using the Ampure XP Reagent (Beckman Coulter), and DNA libraries were prepared for MiSeq sequencing following the manufacturer's protocol. The DNA library was subjected to testing using a MiSeq Reagent Kit with a 500 Cycles V2 Kit (2 × 500 bp) (Illumina, San Diego, CA, USA). The obtained fastq.gz files were preprocessed using QIIME2 v2021.4 and its plugins. Quality filtration, denoising, merging of paired-end reads, and chimera removal were performed using the DADA2 tool with the following setting: denoise-paired --*p*-trim-left-f 20 --*p*-trim-left-r 17 --*p*-trunc-len-f 220 --*p*-trunc-len-r 200. DADA2 processing was performed separately for each MiSeq run with the same parameters, and feature tables and sequences were merged. Alpha and β diversities were assessed using core metric analysis. Alpha rarefaction curves were generated at a sampling depth of 10,000 with ten iterations per depth. Subsequently, the feature sequence was classified using the feature classifier plug-in classify-sklearn. Classification of bacterial 16S rRNA gene sequences was performed using the Silva version 138_1 database, and a phylogenetic tree was constructed using FastTree.

### Sorting of Eub338^+/–^ bacteria and calculation of Eub338 index

To investigate the microbial composition of both viable and nonviable bacteria in fecal samples, we sorted Eub338^+^ and Eub338^–^ bacteria from FISH-labeled fecal samples using a FACSaria fusion cell sorter (BD Biosciences, Franklin Lakes, NJ, USA). More than 50,000 cells were collected in 500 µL of 0.5% FBS/PBS containing with SYTO BC (1:6000, Thermo Fisher). Subsequently, obtained samples were immediately centrifuged at 20,000 × *g* for 5 min at 4 °C, finally the resulting was suspended in 200 μL PBS and stored at −80 °C until DNA extraction. Subsequent steps involved 16S rRNA gene sequencing and microbial community analysis using the aforementioned protocol.^34^^,^^65^ Taxonomic relative abundances obtained from this NGS analysis were then used to calculate the Eub338 index. The relative abundance of bacterial taxa in Eub338^+^ and Eub338^–^ bacteria, and the Eub338 index (EI) was calculated as follows:

E338P_TA_ = sorted Eub338^+^ fraction_taxon abundances_ × frequency of Eub338^+^ bacteria,

E338N_TA_ = sorted Eub338^–^ fraction_taxon abundances_ × frequency of Eub338^–^ bacteria,

EI = − [{log(E338P_TA_)} − {log(E338N_TA_)}]/[{log(E338P_TA_)} + {log(E338N_TA_)}].

Only taxa detected in ≥50% of participants were included in the analysis. Zero values were replaced with half of the minimum nonzero abundance observed across samples, following standard practice in compositional data analysis. The calculation framework was adapted from previously established IgA index methodologies used for sorting-based microbial enrichment analyses.^34^^,^^65^

### Measurement of T84 TEER with fecal supernatant

T84 cells were cultured with DMEM/F12 (Thermo Fisher Scientific) supplemented with 10% FBS and 1% penicillin–streptomycin (Thermo Fisher Scientific). To establish a monolayer, cell culture inserts with a 0.4-μm pore filter membrane (Corning, Corning, NY, USA) were placed onto 24-well plates, and 500 µL of T84 cell suspension (1.5 × 10^5^ cells/mL) was added to each insert. The lower layer of 24-well plates was filled with 1 mL of medium, which was replaced every 2–3 d, and the cells were cultured at 37 °C for 6–10 d.

For the preparation of fecal supernatants from older individuals, 5000 µL of ten-fold diluted fecal samples were centrifuged at 20,000 × *g* for 5 min at 4 °C, and the supernatant was sterilized using a 0.22-µm filter. For the upper insert, 2500 µL of culture medium was removed and replaced with 2500 µL of culture medium containing with fecal supernatant, which consisted of 250 µL of 10 × RPMI1640, 250 µL of FBS, 2.50 µL of penicillin‒streptomycin, 1000 µL of filter-sterilized ten-fold diluted fecal supernatant, and 97.50 µL of sterile water. As a positive control, IFN-γ (40 ng/mL) and TNF-α (50 ng/mL) were added to the lower wells. After 72 h, transepithelial electrical resistance (TEER) values were measured using two electrodes of a Mill-cell ERS (Millipore), one in the upper insert and the other in the lower well. The results were calculated as relative values (%) with respect to the TEER value before the addition of fecal supernatant, which was defined as the TEER value in this report.

### Quantification and statistical analysis

Data were analyzed using GraphPad Prism 8 (GraphPad Software, San Diego, CA, USA). To compare the two groups, *p*-values were determined using the unpaired two-tailed Student's *t* test or the Mann–Whitney *U* test. For the comparison involving more than two groups, Dunnett's multiple comparison test was used to calculate significant values in the starvation culture test with intestinal bacteria, while the Kruskal–Wallis test with Dunn's multiple comparison post-test used for the UC patient data. Correlation analysis was performed using Spearman's correlation analysis in GraphPad Prism 8, and the correlation matrix was corrected using the Benjamini–Hochberg false discovery rate correction. Statistical significance was defined as *p* < 0.05, very significant at <0.01, highly significant at <0.001. Detailed information for each experiment is provided in the figure legends.

## Supplementary Material

Supplemental Table.docx Supplemental Table.docx

Supplemental Material Supplemetal_Figure_1.jpg

Supplemental Material Supplemetal_Figure_2.jpg

Supplemental Material Supplemetal_Figure_3.jpg

Supplemental Material Supplemetal_Figure_4.jpg

Supplemental Material Supplemetal_Figure_5.jpg

## Data Availability

The 16S rRNA-gene amplicon data have been deposited into the NCBI Sequence Read Archive (SRA) database under BioProject ID number PRJNA1076326. This study did not generate any original code. Additional information required to reproduce or reanalyze the data is available upon reasonable request.
