# Supplementary material for: Viable gut bacterial metrics associated with intestinal eubiosis and dysbiosis
Source: Gut Microbes Rep. 2026 Mar 21;3(1):2646054. doi: 10.1080/29933935.2026.2646054 (PMC13034633; doi:10.1080/29933935.2026.2646054)
Supplement: Supplemental Table.docx [file KGMR_A_2646054_SM6973.docx]

| Supplemental Table 2, UC patients and Healthy adults’ information | | | | | |
| --- | --- | --- | --- | --- | --- |
|  |  |  |  |  |  |
| Participants | |  | All | Male | Female |
| UC Patients | | (n) | 35 | 17 | 18 |
|  | age, Mean | (years-old) | 50.7 | 49.0 | 52.5 |
|  | Range | (years-old) | 15-76 | 16-76 | 15-76 |
|  | -19 |  | 4 | 1 | 3 |
|  | 20-39 |  | 7 | 3 | 4 |
|  | 40-59 |  | 10 | 5 | 5 |
|  | 60- |  | 14 | 8 | 6 |
|  |  |  |  |  |  |
|  | active |  | 17 | 6 | 11 |
|  | inactive |  | 18 | 11 | 7 |
|  |  |  |  |  |  |
|  | Left sided |  | 13 | 5 | 8 |
|  | Proctitis |  | 8 | 3 | 5 |
|  | Extensive |  | 14 | 9 | 5 |
|  |  |  |  |  |  |
|  | Mayo Disease Score |  |  |  |  |
|  | 8 |  | 3 | 1 | 2 |
|  | 7 |  | 0 | 0 | 0 |
|  | 6 |  | 3 | 2 | 1 |
|  | 5 |  | 4 | 2 | 2 |
|  | 4 |  | 2 | 0 | 2 |
|  | 3 |  | 5 | 1 | 4 |
|  | 2 |  | 4 | 4 | 0 |
|  | 1 |  | 3 | 2 | 1 |
|  | 0 |  | 11 | 5 | 6 |
|  |  |  |  |  |  |
|  | Medication |  |  |  |  |
|  | 5-ASA |  | 31 | 14 | 17 |
|  | Corticosteroids |  | 5 | 1 | 4 |
|  | AZA |  | 4 | 2 | 2 |
|  | IFX |  | 2 | 2 | 0 |
|  | ADA |  | 1 | 1 | 0 |
|  | GLM |  | 1 | 0 | 1 |
|  | CAP |  | 1 | 0 | 1 |
|  |  |  |  |  |  |
| Healthy adults | | (n) | 25 | 14 | 11 |
| Medication: Medication status at stool collection | | | | |  |
| 5-ASA: 5-aminosalicyclic acid preparation; AZA: Azathioprine. | | | | | |
| IFX: Infliximab; ADU: Adalimumab; GLM: Golimumab. | | | | |  |
| CAP: Leukocytapheresis. | | |  |  |  |

| Supplemental Table 4, participants’ information | | | | | |
| --- | --- | --- | --- | --- | --- |
|  |  |  |  |  |  |
| Participants | |  | All | Male | Female |
| Healthy adult (n) | |  | 93 | 67 | 26 |
|  | Age, Mean (years-old) |  | 38.1 | 39.2 | 35.1 |
|  | Range (years-old) |  | 23 - 58 | 25 - 58 | 23 - 48 |
| Older adults (n) | |  | 109 | 23 | 86 |
|  | Age, Mean (years-old) |  | 88.4 | 87.7 | 88.6 |
|  | Range (years-old) |  | 71 - 100 | 75 - 97 | 71 - 100 |

| Supplemental Table 5, intestinal bacterial strain list | | | | | | | | | | |
| --- | --- | --- | --- | --- | --- | --- | --- | --- | --- | --- |
|  |  |  |  |  |  |  |  |  |  |  |
|  | **Strain** |  | **Abbreviation** |  | **YIT** |  | **Cultivation condition** | | | |
|  | *Phocaeicola vulgatus* |  | *P. vulgatus* |  | 6159^T^ |  | mGAM, Mix gas, 16h | | |  |
|  | *Bacteroides uniformis* |  | *B. uniformis* |  | 6164^T^ |  | mGAM, Mix gas, 16h | | |  |
|  | *Dorea formicigenerans* |  | *D. formicigenerans* |  | 10093^T^ |  | mGAM*, Mix gas, 16h | | |  |
|  | *Clostridium leptum* |  | *C. leptum* |  | 6169^T^ |  | mGAM*, Mix gas, 16h | | |  |
|  | *Bifidobacterium longum* |  | *B. longum* |  | 4037^T^ |  | mGAM*, Mix gas, 16h | | |  |
|  | *Escherichia coli* |  | *E. coli* |  | 6044^T^ |  | mGAM*, Mix gas, 16h | | |  |
|  |  |  |  |  |  |  |  |  |  |  |
|  | Mix gas (N_2_ 90%, H_2_ 5%, CO_2_ 5%) |  |  |  |  |  |  |  |  |  |
|  | * Supplemented with 1%Glucose |  |  |  |  |  |  |  |  |  |
