# Supplementary figures and images for: Viable gut bacterial metrics associated with intestinal eubiosis and dysbiosis
Source: Gut Microbes Rep. 2026 Mar 21;3(1):2646054. doi: 10.1080/29933935.2026.2646054 (PMC13034633; doi:10.1080/29933935.2026.2646054)

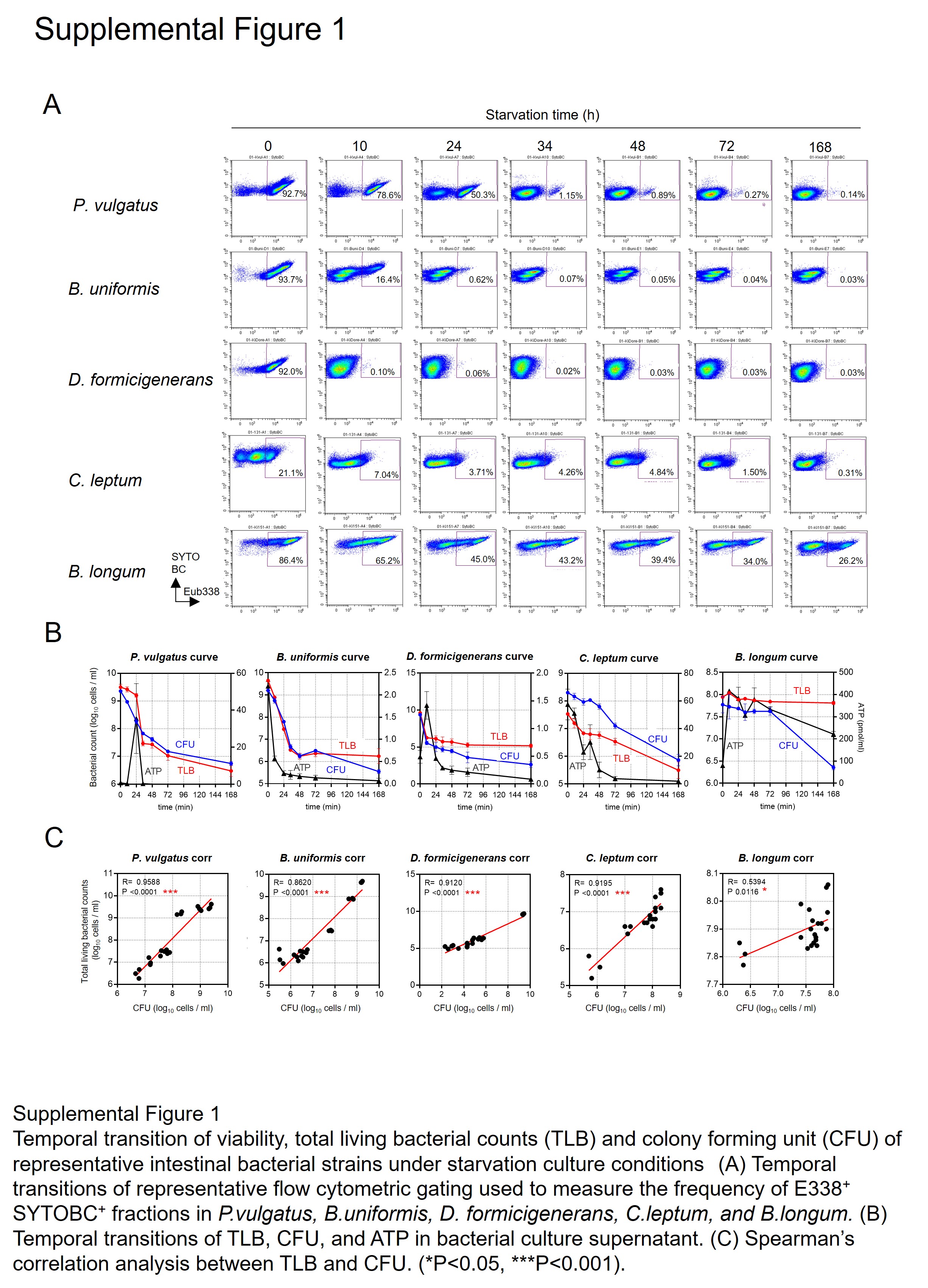

Supplement: Supplemental Material — Supplemetal_Figure_1.jpg [file KGMR_A_2646054_SM3138.jpg]

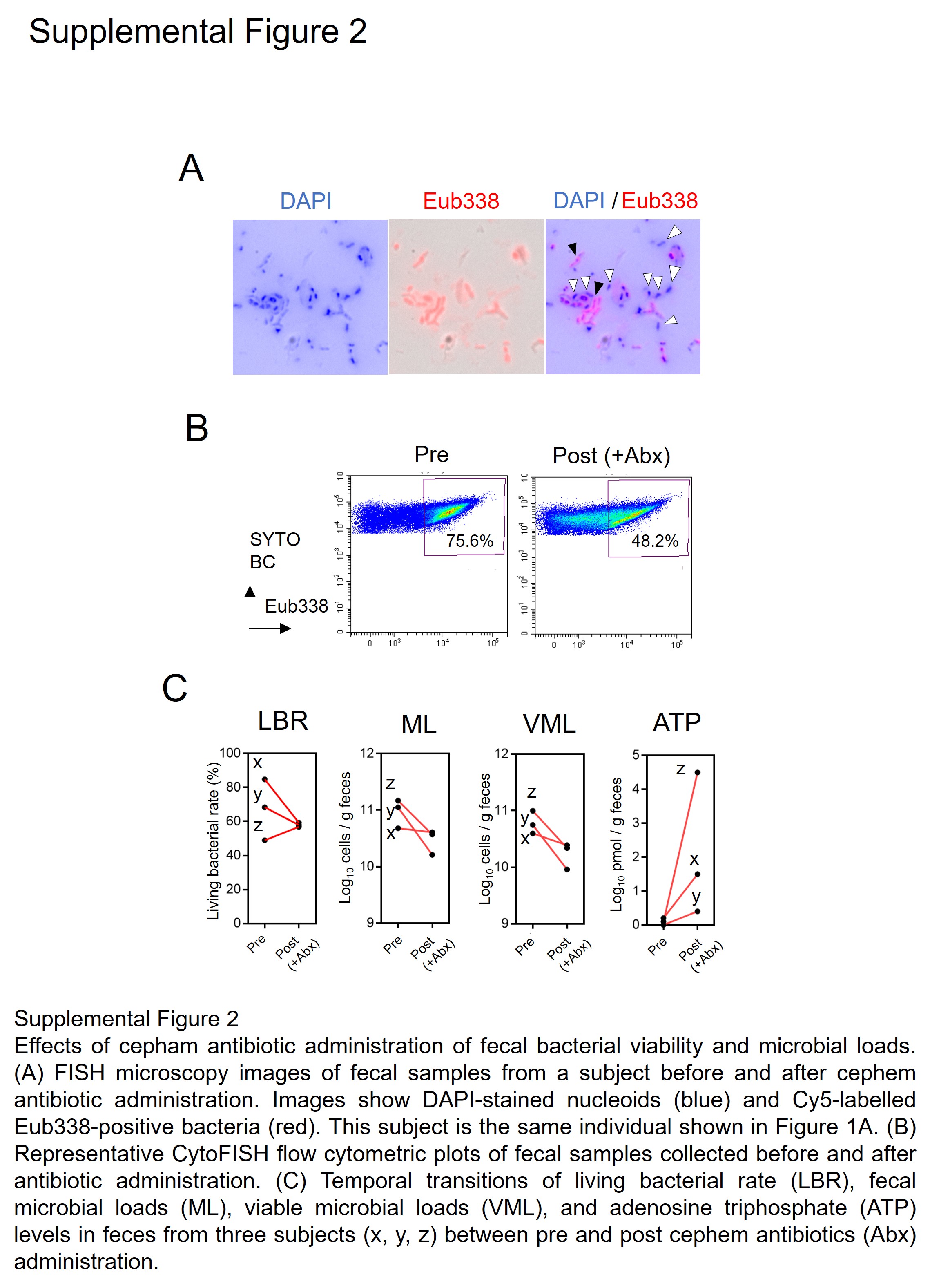

Supplement: Supplemental Material — Supplemetal_Figure_2.jpg [file KGMR_A_2646054_SM3143.jpg]

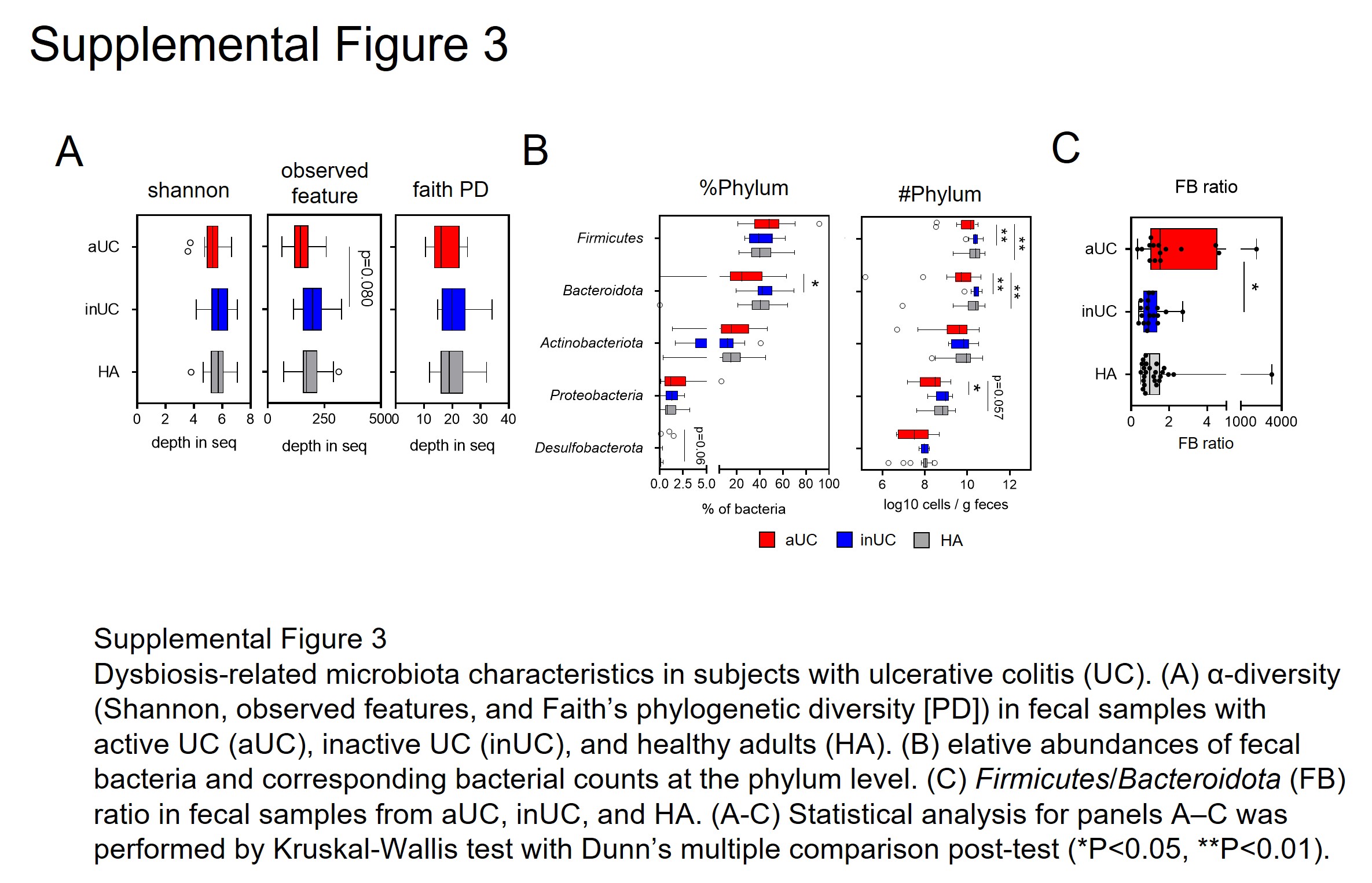

Supplement: Supplemental Material — Supplemetal_Figure_3.jpg [file KGMR_A_2646054_SM3144.jpg]

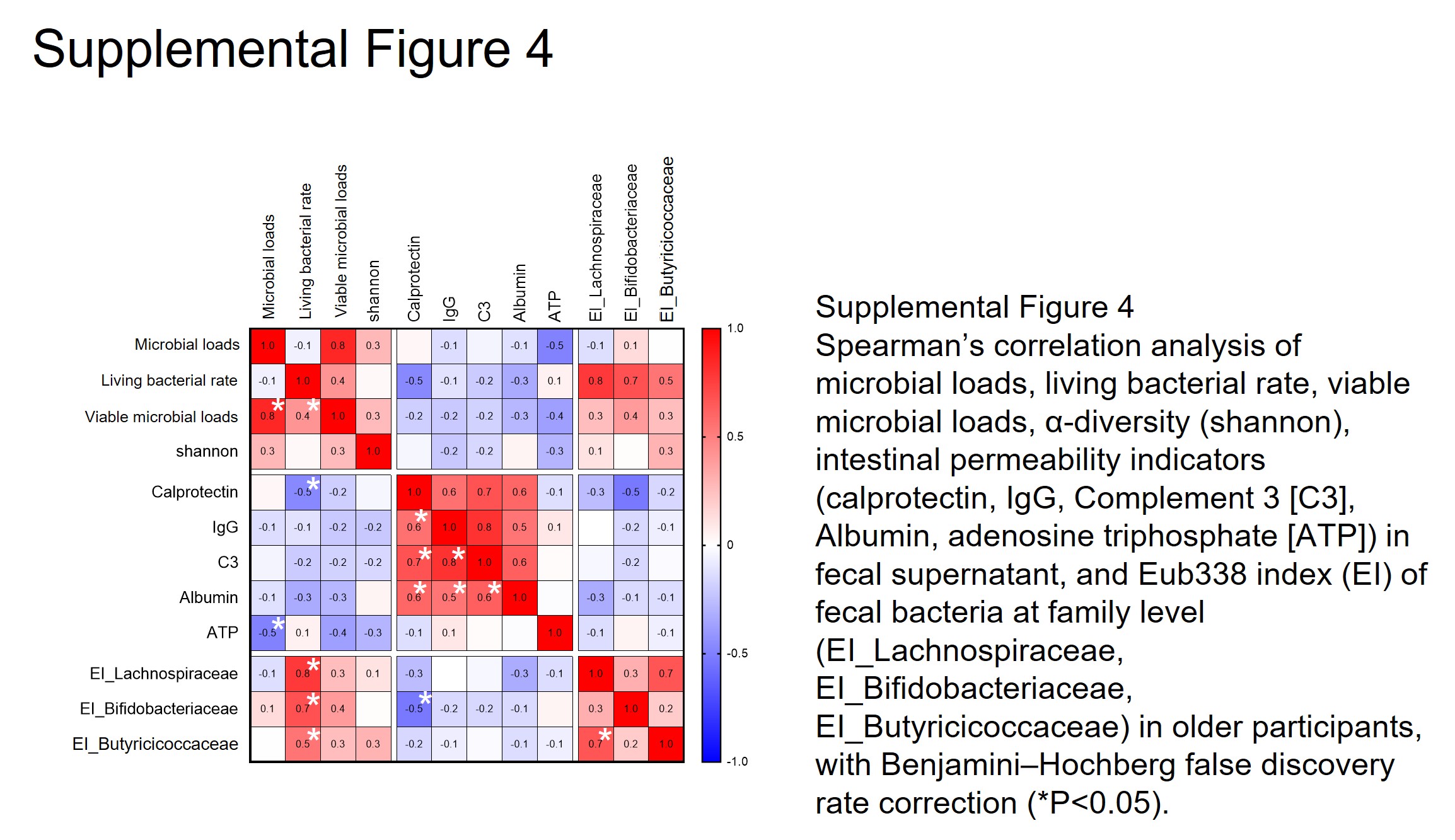

Supplement: Supplemental Material — Supplemetal_Figure_4.jpg [file KGMR_A_2646054_SM3145.jpg]

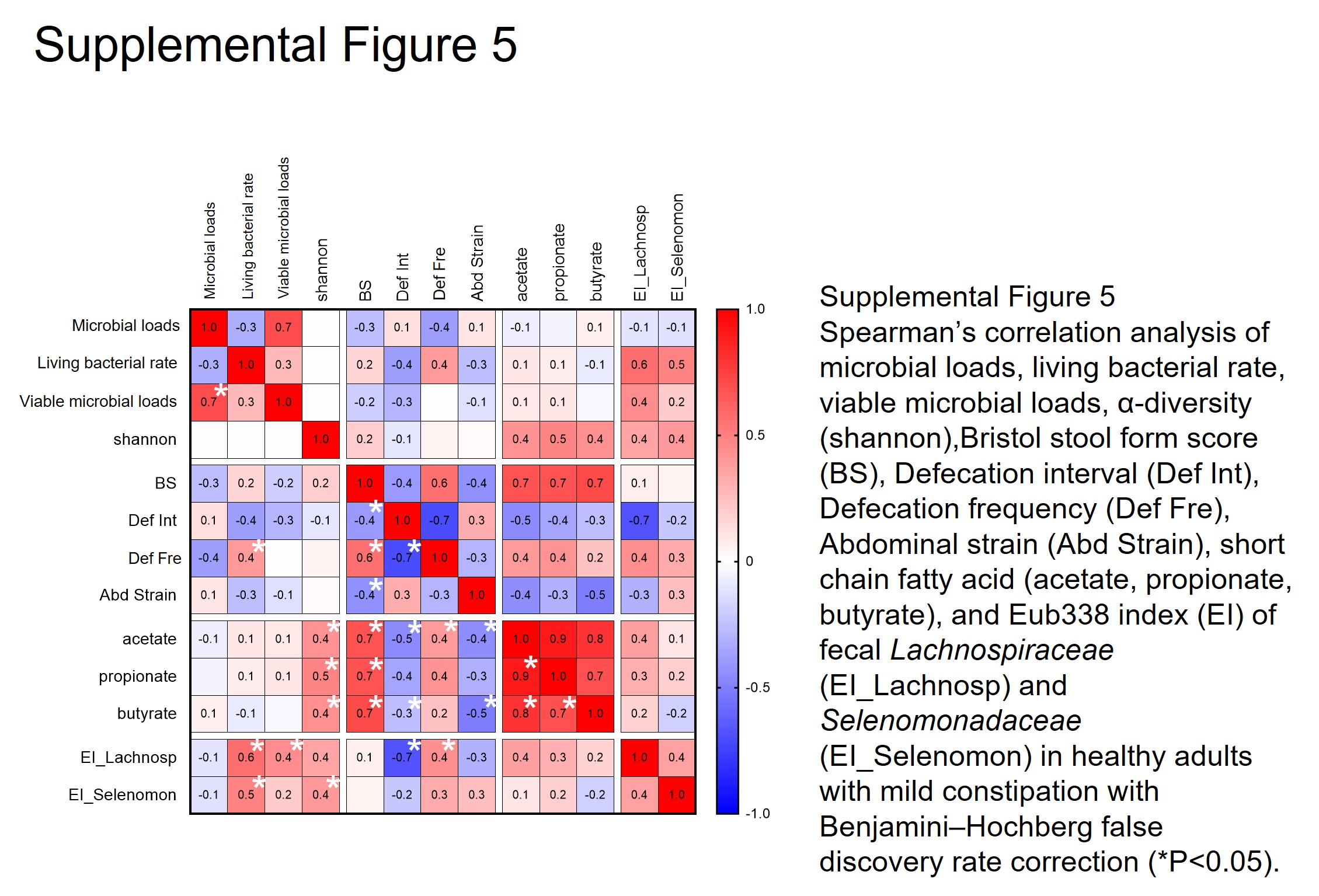

Supplement: Supplemental Material — Supplemetal_Figure_5.jpg [file KGMR_A_2646054_SM3146.jpg]
